# Evaluation of the Novel Sepsis Biomarker Host-Derived Delta-like Canonical Notch Ligand 1—A Secondary Analysis of 405 Patients Suffering from Inflammatory or Infectious Diseases

**DOI:** 10.3390/ijms24119164

**Published:** 2023-05-23

**Authors:** Tobias Hölle, Patrick Rehn, Konstantinos Leventogiannis, Antigone Kotsaki, Theodora Kanni, Nikolaos Antonakos, Christos Psarrakis, Georgia Damoraki, Judith Schenz, Felix C. F. Schmitt, Florian Uhle, Markus A. Weigand, Evangelos J. Giamarellos-Bourboulis, Maximilian Dietrich

**Affiliations:** 1 Department of Anesthesiology, Heidelberg University Hospital, Im Neuenheimer Feld 420, 69120 Heidelberg, Germany; tobias.hoelle@med.uni-heidelberg.de (T.H.);; 2 4th Department of Internal Medicine, Medical School, National and Kapodistrian University of Athens, 11527 Athens, Greece

**Keywords:** sepsis, systemic infection, biomarker, diagnostics, Delta-like Canonical Notch Ligand 1, DLL1, DLL-1

## Abstract

Sepsis is defined as organ failure caused by dysregulated host response to infection. While early antibiotic treatment in patients with acute infection is essential, treating non-infectious patients must be avoided. Current guidelines recommend procalcitonin (PCT) to guide discontinuation of antibiotic treatment. For initiation of therapy, there is currently no recommended biomarker. In this study, we evaluated Host-Derived Delta-like Canonical Notch Ligand 1 (DLL1), a monocyte membrane ligand that has shown promising results in differentiating infectious from non-infectious critically ill patients. Soluble DLL1 levels were measured in plasma samples of six different cohorts. The six cohorts comprise two cohorts with non-infectious inflammatory auto-immune diseases (Hidradenitis Suppurativa, Inflammatory Bowel Disease), one cohort of bacterial skin infection, and three cohorts of suspected systemic infection or sepsis. In total, soluble DLL1 plasma levels of 405 patients were analyzed. Patients were divided into three groups: inflammatory disease, infection, and sepsis (defined according to the Sepsis-3 definition), followed by the evaluation of its diagnostic performance via Area Under the Receiver Operating Characteristics (AUROC) analyses. Patients of the sepsis group showed significantly elevated plasma DLL1 levels compared to patients with uncomplicated infections and sterile inflammation. However, patients with infections had significantly higher DLL1 levels than patients with inflammatory diseases. Diagnostic performance was evaluated and showed better performance for DLL1 for the recognition of sepsis (AUC: 0.823; CI 0.731–0.914) than C-reactive protein (AUC 0.758; CI 0.658–0.857), PCT (AUC 0.593; CI 0.474–0.711) and White Blood Cell count (AUC 0.577; CI 0.46–0.694). DLL1 demonstrated promising results for diagnosing sepsis and was able to differentiate sepsis from other infectious and inflammatory diseases.

## 1. Introduction

Sepsis is defined as organ failure caused by a dysregulated host response to systemic infection [1]. Despite intensive research over the last decades, mortality from septic syndrome is still high. The prognosis depends decisively on early recognition and consecutive induction of specific therapy. For antibiotic treatment, a 4% increase for every hour of delay has been shown [2]. However, it is difficult to distinguish sepsis, especially in the early phase, from other causes of organ failure due to the non-specificity of symptoms. More than one-third of patients initially diagnosed with sepsis turn out to have a noninfectious disease as the true cause of their clinical condition [3]. In these cases, the use of an antibiotic is unnecessary at best or even harmful in case of side effects or the development of resistances. The 2021 Surviving Sepsis Guidelines recommend administering antibiotics immediately in patients with possible septic shock or high likelihood for sepsis. For patients with possible sepsis without shock, the guidelines offer a 3 h timeframe to further assess the likelihood of infectious versus non-infectious causes of acute illness. However, even sepsis patients without shock have a considerable mortality, so that the earliest possible start of therapy is essential [4]. Accordingly, the identification of biomarkers and development of techniques for the rapid recognition of sepsis is one of the top research priorities in sepsis [5].

Soluble host-derived Delta-like Canonical Notch Ligand 1 (DLL1) is a novel, promising and potentially useful biomarker for the diagnosis of sepsis [6]. The notch signaling pathway, which includes DLL1 as a key trans-membranous ligand for the notch receptor, contributes to manifold vital cell functions [7]. Among other consequences, upregulation of this pathway leads to marked excretion of proinflammatory cytokines. In vitro bacterial infection led to an upregulation of DLL1 on the surface of primary human monocytes [8]. Given the importance of monocytes in host response to infection, it seems plausible that soluble DLL1 could be a plausible early biomarker for detecting sepsis. Compared with leucocytes (WBC), C-reactive protein (CRP), or procalcitonin (PCT), a secondary analysis revealed a better discrimination of sepsis from noninfectious systemic inflammatory processes induced by trauma or surgery based on soluble DLL1 [6,9]. So far, data on DLL1 for the diagnostics of sepsis are scarce. The aim of this secondary analysis is to evaluate the performance of DLL1 for the differentiation between sepsis and non-septic inflammatory diseases. Therefore, we measured DLL1 in plasma samples of prospective cohorts with sepsis, signs of infection in the emergency department, bacterial skin infection with *Staphylococcus aureus*, Hidradenitis Suppurativa, and inflammatory bowel disease [10,11,12,13,14,15].

## 2. Results

### 2.1. Baseline Characteristics

**Figure 1:**
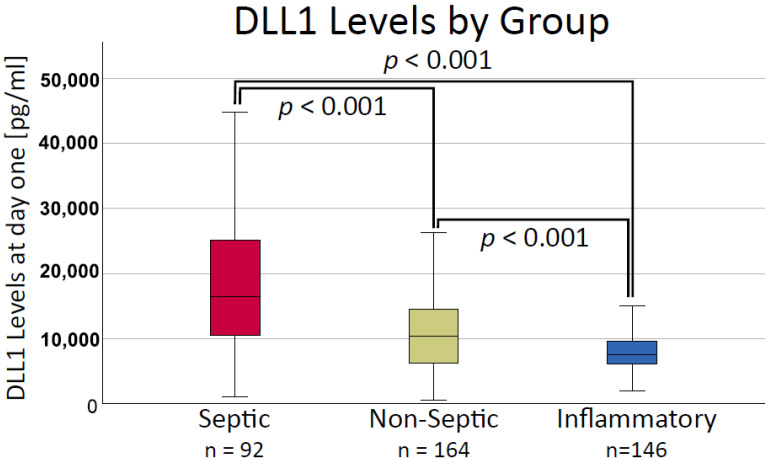
Soluble Delta-like Canonical Notch Ligand 1 (DLL1) plasma concentration in the cohorts divided by etiology. Statistical analysis was performed with a Kruskal–Wallis test and post hoc pairwise comparison using a Mann–Whitney U test. Reported *p*-values are pairwise comparison using the Bonferroni method. A *p*-value < 0.05 was considered significant. [pg/mL]: picogram per milliliter.

DLL1 was measured in plasma samples from 405 patients of six prospectively sampled cohorts (Supplementary Table S1, Supplementary Figure S1). Patients were allocated into three different groups (see Section 4.3): Septic Group (SG), Non-Septic Infection Group (NSIG) and Inflammatory Disease Group (IDG). A total of 93 patients were assigned to the SG, 166 to the NSIG, and 146 to the IDG (Table 1). For more information on cohort baseline characteristics and individual assignment, see Supplementary Information. Age was similar between the SG and NSG, and patients in the IDG were significantly younger than patients in the infectious groups. Sex distribution between the groups was comparable. The Sequential Organ-Failure Assessment (SOFA) Score and APACHE II score were significantly higher in the SG compared to the NSIG and IDG. PCT, CRP and WBC levels for the inflammatory cohorts were not routinely assessed, leading to low numbers of available data in the IDG (n = 6). PCT levels were comparable between groups; CRP and WBC were significantly higher in the SG compared to the NSIG (Table 1). DLL1 was higher in the SG (16,443 8 pg/mL; IQR (10,486–25,101)) compared to NSIG (10,302 pg/mL; IQR (6250; 14,530)) and IDG (7483 pg/mL; IQR (6009; 9621). The NSIG had significantly higher DLL1 values than the IDG (Figure 1).

### 2.2. DLL1 Showed Moderate Correlation with Inflammatory Biomarkers and Weak Correlation with Disease Severity

For the correlation analysis, available data from all timepoints were pooled, and the correlation of DLL1 with established biomarkers for inflammation and disease severity was assessed. DLL1 correlated moderately with PCT (0.492; *p* < 0.001; n = 327) and IL-6 (0.454; *p* < 0.001; n = 274) while only weakly with CRP (0.306; *p* < 0.001; n = 409). An analysis of SOFA and APACHE II scores showed a weak to moderate positive correlation with DLL1 (SOFA: 0.341; *p* < 0.001; n = 477; APACHE II: 0.434; *p* < 0.001; n = 324). Analysis of measurements of day 1 with disease severity revealed a stronger correlation. DLL1 measurements on day 1 showed the strongest correlation with SOFA and APACHE II scores (SOFA: 0.594; *p* < 0.001; n = 164; APACHE II: 0.460; *p* < 0.001; n = 106). Other inflammatory biomarkers showed moderate to weak correlations with DLL1 on day 1, except WBC did not correlate (PCT: 0. 437; *p* < 0.001; n = 113; IL-6: 0.401; *p* = 0.007; n = 44; CRP: 0.281; *p* < 0.001; n = 201; WBC 0.018; *p* = 0.77; n = 257).

### 2.3. DLL1 Level Changes over Time

**Figure 2:**
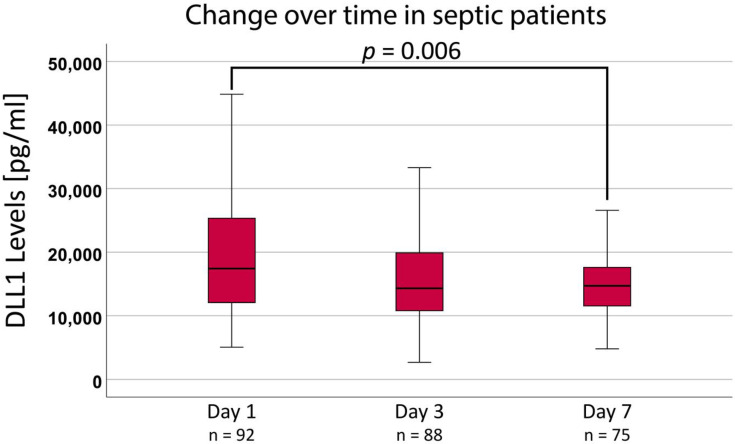
Soluble Delta-like Canonical Notch Ligand 1 (DLL1) plasma concentration and the change over time in septic patients. Statistical analysis was performed using a Friedman test. Reported *p*-values are adjusted using the Bonferroni method. A *p*-value < 0.05 was considered significant. [pg/mL]: picogram per milliliter.

DLL1 values in septic patients stayed elevated over time. A significant decrease compared to initial values was observed only after 7 days (Figure 2).

### 2.4. Diagnostic Performance of DLL1 for Sepsis

**Figure 3:**
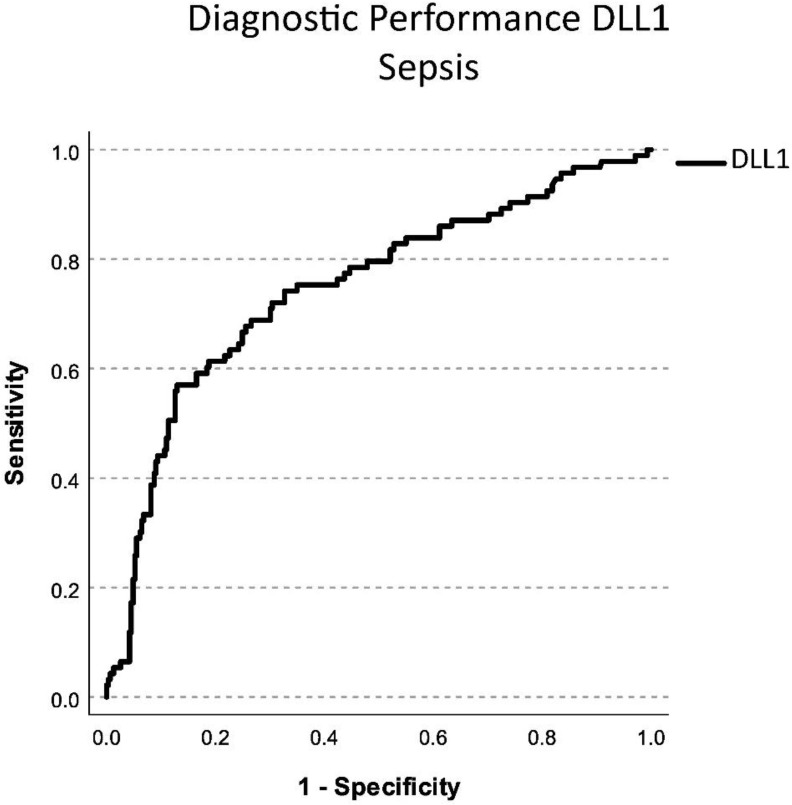
Diagnostic performance in differentiating septic patients to infectious or inflammatory disease of soluble Delta-like Canonical Notch Ligand 1 (DLL1) via AUROC analysis. The *x*-axis shows the sensitivity with which a biomarker can detect the presence of sepsis. The *y*-axis shows 1-the specificity with which a biomarker can detect sepsis. Diagnostic performance was determined according to the area under the curve.

We analyzed DLL1 values at baseline and the diagnostic performance via an Area Under the Receiver Operating Characteristics (AUROC) analysis of all patients from which a DLL1 value was available (n = 402). DLL1 showed significant diagnostic performance for differentiating sepsis (area under the curve (AUC) 0.746; CI 0.685–0.807) from non-septic infections and inflammatory diseases (Figure 3). To compare the performance of DLL1 to other biomarkers of inflammation and sepsis, an analysis of patients with DLL1, PCT, WBC, and CRP available at baseline (n = 92; 51 septic patients, 41 infectious/non-infectious patients) was performed.

**Figure 4:**
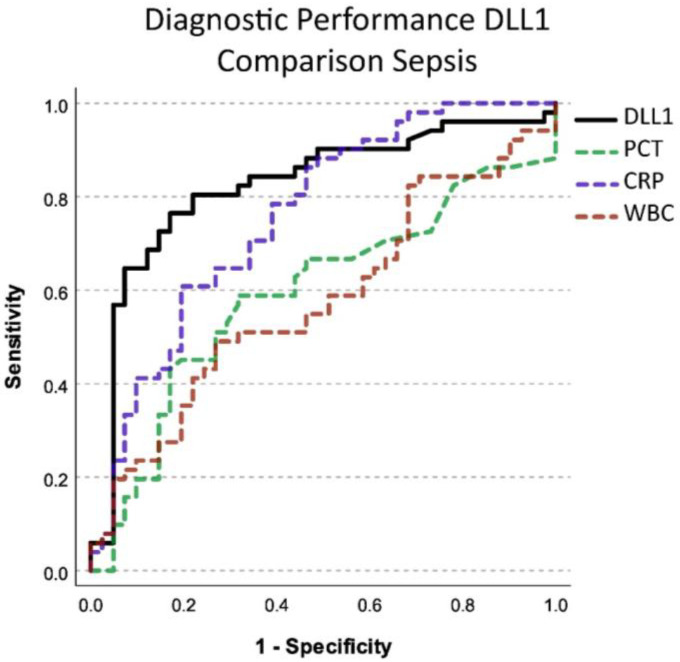
Diagnostic performance in differentiating septic from non-septic patients using soluble Delta-like Canonical Notch Ligand 1 (DLL1) compared to other established biomarkers for infection. PCT: Procalcitonin, CRP: C-reactive protein, WBC: White blood cell count. The *x*-axis shows the sensitivity with which a biomarker can detect the presence of sepsis. The *y*-axis shows 1–the specificity with which a biomarker can detect sepsis. Diagnostic performance was determined according to the area under the curve.

Post hoc diagnostic performance of DLL1 for the recognition of sepsis was 0.823 (CI 0.731–0.914) with a cut-off at 10,623 µg/mL compared to CRP (cut-off 125.6 mg/l; AUC 0.758; CI 0.658–0.857), PCT (cut-off 0.22 ng/mL; AUC 0.593; CI 0.474–0.711), and WBC (cut-off 12,480 /µL; AUC 0.577; CI 0.46–0.694) (Figure 4). PCT and WBC diagnostic performance did not reach significant diagnostic prediction in this analysis. Sensitivity for these cut-offs was at 0.765 for DLL1, 0.608 for CRP, 0.588 for PCT, and 0.490 for WBC. Specificity was at 0.829 for DLL1, 0.805 for CRP, 0.683 for PCT, and 0.732 for WBC.

### 2.5. Prognostic Performance of DLL1 in Sepsis

**Figure 5:**
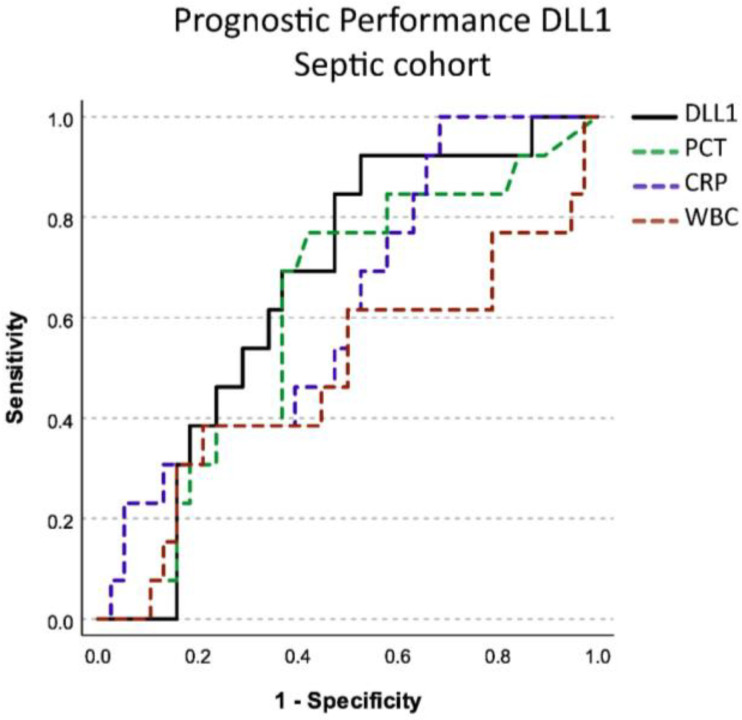
Figure shows prognostic performance in predicting 28-day mortality in septic patients of soluble Delta-like Canonical Notch Ligand 1 (DLL1) compared to other established biomarkers for infection on day 1. PCT: procalcitonin, CRP: C-reactive protein, WBC: white blood cell count. The *x*-axis shows the sensitivity with which a biomarker can predict 28-day mortality. The *y*-axis shows 1–the specificity with which a biomarker predict death. Prognostic performance was determined according to the area under the curve.

Prognostic prediction of 28-day mortality was analyzed for all four biomarkers on day 1 in the septic cohort. There was a small number of data available for this analysis (n = 13 non-survivors, n = 38 survivors). DLL1 was the only biomarker with a small but significant predictiveness in this model (AUC 0.662; CI 0.506–0.818), and the best predictive cut-off was 13.830 µg/mL. Other biomarkers did not show good predictive value for 28-day mortality (Figure 5). Sensitivity for prediction of mortality in this analysis was 0.923 with a specificity of 0.474.

## 3. Discussion

Investigation of new diagnostics and biomarkers for the early recognition of sepsis is one of the top research priorities defined by the Surviving Sepsis Campaign expert panel [5]. Soluble DLL1 is a promising new biomarker, produced among other sources by monocytes in response to bacterial infection [8]. There have been studies describing DLL1 diagnostic performance for infectious and inflammatory diseases such as meningitis, COPD, or congestive heart failure [16,17]. The diagnostic potential of DLL1 has successfully been demonstrated in sepsis research [6,9]. Decker et al. were able to show the applicability of DLL1 in infections or complicative courses in immunosuppressed patients after liver transplantation. To our knowledge, Hildebrand et al. were the first who investigated DLL1 for the diagnosis of sepsis. The diagnostic performance of DLL1 in sepsis was confirmed by the work of Schneck et al. Unfortunately, both studies compared patients with septic shock against patients after surgical trauma as a control group. We measured DLL1 levels in plasma samples of six patient cohorts with infectious and inflammatory diseases to investigate the biomarker’s ability to differentiate between sterile and pathogen-induced inflammation. Patients with autoinflammatory diseases (HS, IBD) showed significantly lower DLL1 levels than patients with infection. Further patient cohorts with infection were separated into septic and non-septic. Septic patients had significantly higher DLL1 levels than infectious patients without organ failure. These findings are in line with the results of previous studies [6,18]. However, to our knowledge, this study is the first assessing the ability of DLL1 to differentiate between infectious, inflammatory, and septic patients. Especially in these groups, the identification of septic patients with established biomarkers has proven difficult.

Originally, DLL1 was known from cancer research. It serves as a ligand for the Notch signaling pathway, which is important for tissue differentiation and angiogenesis [18]. Only recent findings indicated a role for DLL1 in the pathophysiology of infections. Moll et al. could show that the increase of DLL1 was accompanied by a loss of endothelial barrier function [19]. It is already known that neutrophil activation and loss of endothelial function play an essential role in the pathophysiology of sepsis [20]. This offers a possible explanation why DLL1 is elevated in septic patients in particular and why it is suitable as a biomarker.

DLL1 correlated moderately with established inflammatory biomarkers such as PCT and CRP. Therefore, DLL1 may provide additional information to CRP and PCT in infectious disease diagnostics. The difference between the overall correlation and the correlation on day one can be explained by the different kinetics of the biomarkers. In contrast to classical biomarkers such as CRP and PCT, DLL1 decreases more slowly, this discrepancy explains a decreasing correlation of the values in the course. In parallel, the better correlation at baseline with disease severity can be explained. Since DLL1 remains elevated in proportion even after the severity of the disease has decreased. Hildebrand et al. reported a higher performance of DLL1 in discriminating between sepsis and sterile inflammation induced by severe trauma and surgery [6]. Schneck et al. found similar results in patients with sepsis compared to patients after major abdominal and cardiac surgery [21]. The results of the present analysis support these findings and show that the measurement of DLL1 provides additional diagnostic value to distinguish sepsis from chronic-inflammatory diseases.

The current Surviving Sepsis Campaign guidelines suggest using PCT in addition to clinical evaluation for the decision of when to discontinue antimicrobials. No biomarker is recommended to guide the decision to initiate antimicrobial therapy [4]. In two cohorts, data on the course of DLL1 in septic patients were available. DLL1 levels remained high and showed a significant response to therapy only after 7 days [6]. Hildebrand et al. showed similar results: DLL1 values remained almost as high as on the day of admission over 1 week [6]. Therefore, DLL1 could be helpful to support the recognition of sepsis and the consecutive decision on when to start sepsis therapy. The delay of decline in DLL1 levels as a sign of successful therapy limits its use for monitoring disease progression.

Clearly, this study has several limitations. As this was a retrospective analysis of different studies, plasma samples were not available for every patient in each cohort. In addition, the cohorts without severe infections lacked additional data on survival and disease severity. Follow-up and longitudinal progression were also only available in the septic cohort. Furthermore, routine data on other biomarkers were not routinely available in the non-critically ill (infectious and inflammatory) cohorts, or on disease severity, which attenuates the results of the comparative analysis.

So far, data on DLL1 for sepsis diagnostics are limited. However, the release of DLL1 by monocytes in response to a bacterial stimulus and existing retrospective data from clinical cohorts suggest that DLL1 is a promising biomarker for diagnosis of sepsis. Further prospective studies are necessary to evaluate DLL1 as a biomarker for systemic infections and sepsis in different cohorts of critically ill patients in the intensive care unit and emergency department.

## 4. Materials and Methods

### 4.1. Study Cohorts

We conducted a secondary combinational analysis through evaluating data from six study cohorts. Before the enrollment, all studies were approved by the responsible ethics committees. For all studies, written informed consent was required from all included patients or their legal representatives if they were unable to give consent themselves.

Cohort 1 (A06-29) [10] consisted of prospectively enrolled patients with Gram-negative bacteremia, intrabdominal infections, or acute pyelonephritis. All participants underwent study treatment of either 1 g of clarithromycin for four days or a placebo in the same manner in addition to the standard antibiotics. Blood samples were drawn at enrollment and on day 28. Exclusion criteria were neutropenia, HIV infection, intake of corticosteroids (≥1 mg/kg equivalent prednisolone for at least 1 month), and the intake of any macrolide. Plasma samples and data of 47 participants were available for analysis. The study was approved by the National Ethics Committee of Greece (EudraCT number: 2006-004886-33; ClinicalTrials.gov reference number: NCT01223690).

Cohort 2 (AIDA) [15] consisted of prospectively enrolled patients with acute bacterial skin and skin structure infection (ABSSSI) caused by MRSA. This trial’s goal was to compare antimicrobial treatment with oral minocycline plus rifampicin to treatment with oral linezolid. Exclusion criteria were diabetic foot syndrome, osteomyelitis, and patients already undergoing antimicrobial treatment. Plasma samples and data of 86 participants were available for analysis. The study was approved by the National Ethics Committee of Greece (EudraCT number: 2014-001276-56).

Cohort 3 (PROMPT) [11] consisted of prospectively enrolled patients with any sign of probable infection who were admitted to the Emergency Department. Blood samples were drawn within the first hour of admission into the hospital. Plasma samples and data of 86 participants were available for analysis. The local Institutional Ethics Committees approved the study (ClinicalTrials.gov reference number: NCT03295825).

Cohort 4 (ACA-GREC) [12] consisted of prospectively enrolled patients suffering from ventilator-associated pneumonia. Study treatment included either an additional 1 g of Clarithromycin for 3 days or a placebo for 3 days in addition to the standard antibiotics. Blood samples were collected before starting the study treatment and for six consecutive days thereafter. No exclusion criteria were applied. Plasma samples and data of 47 participants were available for analysis. The study was approved by the National Organization for Medicines of Greece (ClinicalTrials.gov reference number: NCT00297674).

Cohort 5 (HS) [13] consisted of prospectively enrolled patients undergoing a study treatment of either MABp1 (antibody targeting IL-1α) or placebo for hidradenitis suppurativa. Blood samples were drawn at the start of the treatment and after 12 weeks. Plasma samples and data of 90 participants were available for analysis. No exclusion criteria were applied. The study was approved by the National Ethics Committee of Greece (reference number: EudraCT number 2015-002321-20; ClinicalTrials.gov reference number: NCT02643654).

Cohort 6 (IBD) [14] consisted of chronic inflammatory bowel disease patients and healthy patients. The aim of this study was to assess the NLRP3 inflammasome activation in these patients. A blood sample was taken at inclusion in this study. Colorectal cancer or cancer of the small intestine, severe systematic disease, current chemotherapy or radiotherapy, and the intake of NSAR or antibiotics were exclusion criteria. Plasma samples and data of 50 participants were available for analysis. The study was approved by the Ethics Review Board of ATTIKON University General Hospital (reference number: 7571/09.11.2011).

### 4.2. Measurement of DLL1

A commercially available enzyme-linked immunosorbent assay (ELISA) was used to measure DLL1 levels (RayBiotech, Peachtree Corners, GA, USA). The samples were prepared according to the manufacturer’s instructions and diluted 30-fold for optimal analysis using the provided assay diluent. Measurements were conducted using an ELx808 microplate reader (BioTek Instruments, Inc., Winooski, VT, USA), and concentration calculation was performed via the corresponding Gen5 software version 2.09 (BioTek Instruments, Inc., Winooski, VT, USA).

### 4.3. Allocation of Study Groups

In total, data of 405 patients were analyzed. Enrolled participants of all studies were then divided into 3 subgroups. Group 1 (Septic Group, SG) contains patients fulfilling the Sepsis-3 consensus criteria: all patients in this group had organ dysfunction defined as a Sequential Organ-Failure Assessment Score (SOFA) ≥ 2 caused by infection [1]. Group 2 (Non-Septic Infection Group, NSIG) includes patients with infection but without organ failure according to Sepsis-3 consensus criteria. Group 3 (Inflammatory Disease Group, IDG) contains patients with autoinflammatory diseases without infection.

### 4.4. Statistical Analysis

Statistical analysis was performed using IBM SPSS Statistics Version 28.0.0.0 (IBM, Armonk, NY, USA). Descriptive statistics and general visualization were performed on all available data. Continuous variables were compared using the Mann–Whitney U and Kruskal–Wallis tests as appropriate and were adjusted using the Bonferroni method. The evaluation of the change over time was carried out with a Friedman test and was also adjusted using the Bonferroni method. If not stated otherwise, data are reported as median and interquartile ranges (IQRs). Categorical values are reported as absolute and relative frequencies, and analysis was performed using the Chi-squared test. For correlation, available data from all timepoints were pooled, and correlation was evaluated using the Spearman Rho test.

The post hoc diagnostic performance of DLL1 for sepsis was assessed via a receiver operating characteristic (ROC) analysis for DLL1 values at day 1 or respective measurements. Results are reported as the area under the curve (AUC) and respective 95% confidence interval (CI) for WBC, PCT1, CRP, and DLL1.

Results were considered significant at a cut-off of 0.05 or below. In the case of AUROC analysis, significance was considered if the 95% CI did not include 0.5.

## 5. Conclusions

DLL1 is a promising biomarker for sepsis diagnostics. It was able to distinguish sepsis from local infection or chronic inflammatory disease. Therefore, the biomarker could be potentially useful for sepsis diagnostics in both medical and surgical intensive care patients. Prospective validation of DLL1 as a sepsis biomarker in larger cohorts is warranted to support the robustness of the promising results to date.

## Figures and Tables

**Table 1:** All study cohorts by disease. Metric data are represented as median and interquartile range; categorial data are reported as absolute and relative frequencies. Statistical analysis of metric parameters was performed with a Kruskal–Wallis test, and categorical values were compared using a Chi-Squared test. A *p*-value < 0.05 was considered significant.

	Septic		Non Septic		Inflammatory		*p*-Value
Count	93	available	166	available	146	available	
Age (years)	68.5 (55 ;76)	93	69 (43 ;79)	146	47.5 (32.5 ;60)	56	<0.001
Sex (male)	32 (34%)	93	68 (41%)	164	30 (54%)	56	0.068
SOFA Score	7 (4 ;10)	93	0.5 (0 ;1)	66	0 (0 ;1)	6	<0.001
APACHE II Score	16.5 (14.5 ;20)	48	6 (2 ;8)	57	7 (7 ;7)	1	<0.001
Height (cm)	-		170 (165 ;180)	83	-		
Weight (kg)	-		70.5 (60.5 ;85)	83	-		
							
WBC (1/µL)	12850 (8490 ;16400)	93	10060 (7130 ;13200)	159	13760 (13090 ;14840)	6	<0.001
PCT (ng/mL)	0.23 (0.05 ;1.38)	71	0.075 (0.05 ;0.4)	38	0.1 (0.08 ;0.15)	5	0.311
CRP (mg/L) day1	143.05 (64.8 ;244)	67	12.535 (4.435 ;44.55)	128	97.65 (6.66 ;175)	6	<0.001
28 day mortality	23 (24.5%)	93	1 (1.3%)	79	0 (0 ;)	6	<0.001

## Data Availability

The datasets used and analyzed during the current study are available from the corresponding author on reasonable request.

## Supplementary Materials

The following supporting information can be downloaded at: https://www.mdpi.com/article/10.3390/ijms24119164/s1.
