# Supplementary material for: Evaluation of the Novel Sepsis Biomarker Host-Derived Delta-like Canonical Notch Ligand 1—A Secondary Analysis of 405 Patients Suffering from Inflammatory or Infectious Diseases
Source: Int J Mol Sci. 2023 May 23;24(11):9164. doi: 10.3390/ijms24119164 (PMC10252395; doi:10.3390/ijms24119164)
Supplement: Supplementary file 1 [file ijms-24-09164-s001.zip › ijms-2356858-supplementary.pdf]

Supplement Information

Baseline characteristics and group allocation

DLL1 was measured in plasma samples from 405 patients of six prospectively sampled cohorts. Patients with Hidradenitis Suppurativa (HS) and Inflammatory Bowel Disease (IBD) were categorised as inflammatory, non-infectious patients (IG). Participants of the AIDA study, consisting of patients with a methicillin-resistant *Staphylococcus aureus* cutaneous infection, were considered infectious, non-septic (NSG). Infectious patients of the PROMPT, ACA-GREC and A06-09 were classified infectious, either septic (SG) or non-septic groups, following the Sepsis-3 definition. The PROMPT study reported no suspected infection in 6 patients, these were allocated to the inflammatory disease group. Baseline values of available data, as well as the number of patients assigned to each group are shown in supplementary table 1. Baseline DLL1 values for the individual cohorts are shown in supplementary figure 1:

Table S1. *Baseline characteristics of the different studies and the subgroups. Patients were classified according to the Sepsis-III definition. Patients with Hidradenitis Suppurativa (HS) and Inflammatory Bowel Disease (IBD) were classified as inflammatory. Data is reported as median and interquartile range. WBD: white blood cell count, PCT: procalcitonin, CRP: C-reactive protein, cm: centimetre, kg: kilogram, [1/µl]: count per microlitre, [ng/ml]: nanogram per microlitre, [mg/ml]: milligram per millilitre.*

	A06-29		ACA-GREC	AIDA	PROMPT			HS	IBD
	Septic	Non septic	Septic	Non septic	Septic	Non septic	Inflammatory	Inflammatory	Inflammatory
Count	44	3	47	86	3	77	6	90	50
Age (years)	73 (62 ;79,5)	71 (44 ;74)	63 (46 ;70)	75 (68 ;82,5)	84 (52 ;92)	50 (36 ;74)	50,5 (37 ;59)	-	46 (32 ;60)
Sex (male)	15 (34%)	1 (33%)	15 (32%)	34 (40%)	2 (67%)	33 (43%)	5 (83%)	-	25 (50%)
SOFA Score	5 (3 ;11)	1 (1 ;1)	7 (6 ;9)		4 (4 ;8)	0 (0 ;1)	0 (0 ;1)		
APACHE II Score			16,5 (15 ;20)		18,5 (9 ;28)	6 (2 ;8)	7 (7 ;7)		
Height (cm)				170 (165 ;180)					
Weight (kg)				70,5 (60,5 ;85)					
WBC (1/µl)	13035 (9135 ;16105)	8830 (6560 ;21350)	12730 (8400 ;16510)	9320 (7100 ;12760)	9120 (2660 ;24700)	10660 (7640 ;13400)	13760 (13090 ;14840)		
PCT (ng/ml)	0,985 (0,14 ;6,035)	0,07 (0,01 ;7,23)	0,07 (0,04 ;0,53)		0,545 (0,12 ;0,97)	0,08 (0,05 ;0,4)	0,1 (0,08 ;0,15)		
CRP (mg/l)	142,7 (94 ;206,6)	232,05 (214,1 ;250)	190,5 (53,5 ;252,5)	9,79 (4,5 ;24,4)	91,6 (27,5 ;117)	17,2 (4,37 ;57,4)	97,65 (6,66 ;175)		
28 day mortality	8 (18,2%)	0 (0)	15 (31,9%)		0 (0)	1 (1,3%)	0 (0)		

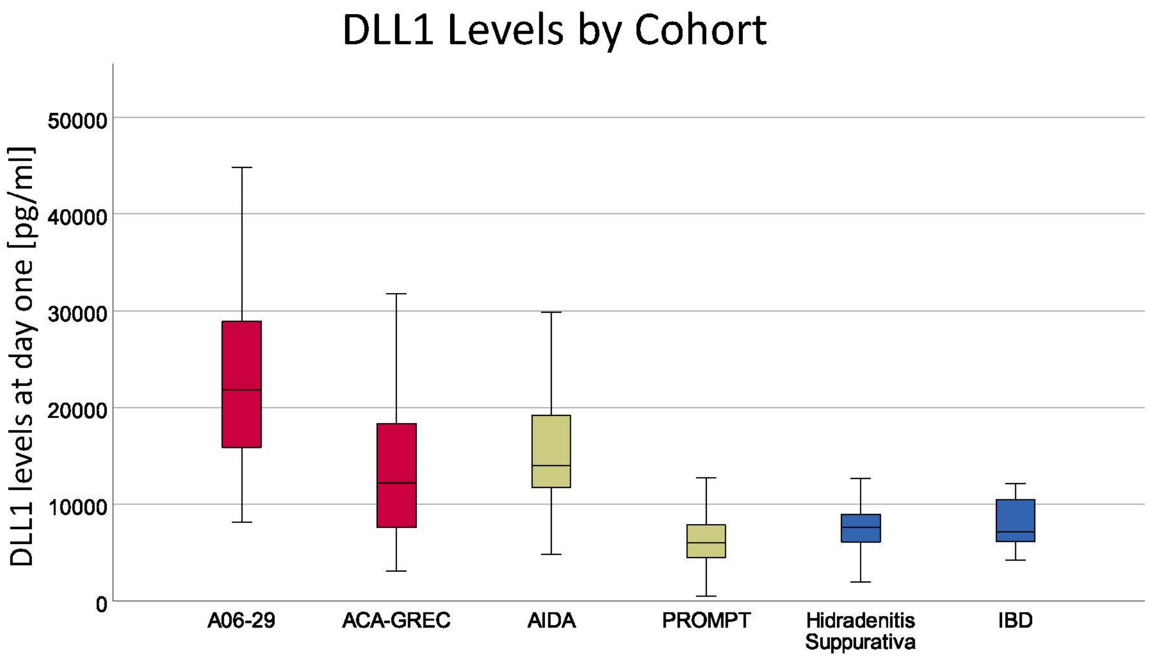

Figure S1. *Delta-like Protein 1 (DLL1) values for the individual cohorts. No group comparison was performed on this data. A06-29 and ACA-GREC cohorts are predominantly septic patients (red), AIDA and PROMPT patients consist mostly of non-septic patients (yellow) and Hidradenitis Suppurativa and Inflammatory bowel disease (IBD) patients were considered inflammatory disease patients (blue). picogram (pg), millilitre (ml).*
